# Evaluation of the Effectiveness of Geogrids Manufactured from Recycled Plastics for Slope Stabilization—A Case Study

**DOI:** 10.3390/polym16081151

**Published:** 2024-04-19

**Authors:** Lenin Vicuña, Ximena Jaramillo-Fierro, Paúl Eduardo Cuenca, Brenda Godoy-Paucar, Jorge Daniel Inga-Lafebre, Jose Luis Chavez Torres, Juan Fernando García, Diana Guaya, Juan Diego Febres

**Affiliations:** 1 Departamento de Producción, Facultad de Ciencias Exactas y Naturales, Universidad Técnica Particular de Loja, San Cayetano Alto, Loja 1101608, Ecuador; lvicuna@utpl.edu.ec (L.V.); pecuenca2@utpl.edu.ec (P.E.C.); jdinga@utpl.edu.ec (J.D.I.-L.); jdfebres@utpl.edu.ec (J.D.F.); 2 Departamento de Química, Facultad de Ciencias Exactas y Naturales, Universidad Técnica Particular de Loja, San Cayetano Alto, Loja 1101608, Ecuador; jfgarcia@utpl.edu.ec (J.F.G.); deguaya@utpl.edu.ec (D.G.); 3 Carrera de Ingeniería Industrial, Facultad de Ciencias Exactas y Naturales, Universidad Técnica Particular de Loja, San Cayetano Alto, Loja 1101608, Ecuador; bngodoy@utpl.edu.ec; 4 Departamento de Ingeniería Civil, Facultad de Ingenierías y Arquitectura, Universidad Técnica Particular de Loja, San Cayetano Alto, Loja 1101608, Ecuador; jlchavez3@utpl.edu.ec

**Keywords:** geogrids, recycled plastics, slope stabilization, HDPE, PP, mechanical properties, SolidWorks, sustainability

## Abstract

This study aimed to investigate the sustainable use of recycled plastics, specifically polypropylene (PP) and high-density polyethylene (HDPE), in the manufacture of geogrids for geotechnical and civil engineering applications. Plastics were collected from a recycling center, specifically targeting containers used for food, cleaning products, and other domestic packaging items. These plastics were sorted according to the Möbius triangle classification system, with HDPE (#2) and PP (#5) being the primary categories of interest. The research methodologically evaluates the mechanical properties of PP/HDPE (0/100, 25/75, 50/50, 75/25 and 100/0% *w*/*w*) composites through tensile and flexural tests, exploring various compositions and configurations of geogrids. The results highlight the superiority of pure recycled HDPE processed into 1.3 mm thick laminated yarns and hot air welded for 20 to 30 s, exhibiting a deformation exceeding 60% in comparison to the PP/HDPE composites. Through SolidWorks^®^ Simulation, it was shown that the adoption of a trigonal geogrid geometry optimizes force distribution and tensile strength, significantly improving slope stabilization efficiency. Based on the results obtained, a laboratory-scale prototype geogrid was developed using an extrusion process. The results underscore the importance of careful composite design and yarn configuration selection to achieve the desired mechanical properties and performance in geogrid applications. It emphasizes the potential of recycled plastics as a viable and environmentally friendly solution for stabilizing slopes, contributing to the reduction in plastic waste and promoting sustainable construction practices.

## 1. Introduction

Slope stabilization is a crucial area of interest in geotechnical engineering, focusing on the reinforcement of earth structures to prevent landslides and structural failures [1]. In this context, artificial reinforcement techniques have gained importance due to their efficacy and versatility [2]. According to the literature, the use of geosynthetics, particularly geogrids, has established itself as an efficient solution for strengthening slopes and mitigating the effects of external disruptions [3,4].

Geogrids, consisting of solid networks of synthetic material, play multiple roles in slope stabilization, including reinforcement, separation, drainage, filtration, protection, and containment [5,6]. Each of these functions is fundamental to improving the overall stability of slopes and optimizing their performance under various environmental and load conditions.

The use of geogrids contributes significantly to the optimization of the use of available land in construction projects, resulting in a notable reduction in costs associated with the transportation and handling of selected fill materials, as well as the management of inadequate fills [7]. Additionally, as part of strategies to improve environmental sustainability, vegetation cover is often implemented on land surfaces stabilized with geogrids. In this way, geosynthetics play a vital role as soil surface stabilizers, while vegetation not only provides aesthetic benefits with its green appearance, but also offers protection against surface erosion [8]. The synergy between geosynthetics and vegetation is vital, as both elements integrate to provide combined benefits essential for the long-term sustainability and conservation of land surfaces. This interaction between geosynthetic and biological components is a key area of study in environmental geotechnical engineering, underscoring its importance for the stability and ecological resilience of construction and land rehabilitation projects [9,10].

The incorporation of recycled materials in geotechnical engineering, especially in the manufacturing of geogrids from recycled plastics, is a practice that has gained relevance in the field of environmental sustainability [11,12]. This strategy addresses several critical aspects related to sustainability, including the reduction in the demand for virgin materials, the minimization of solid waste generation, and the decrease in carbon dioxide emissions associated with the manufacturing of new polymeric materials [13,14,15]. Furthermore, the use of recycled materials in geotechnical engineering is noted for its cost-effectiveness, as it allows the use of inferior quality soils that would otherwise require costly improvement or replacement processes [16,17,18].

The effectiveness of geogrids made from recycled plastic for slope stabilization is well documented through various experimental investigations [19,20,21,22,23]. In fact, many studies have demonstrated that recycled plastics used in geogrids maintain mechanical properties and performance comparable to virgin plastics [24,25]. This is essential, as it ensures that the structural integrity and functionality of the geogrids are not compromised, despite using recycled materials. Thus, geogrids made from recycled plastics are not only viable from a technical perspective but also represent a sustainable and environmentally friendly solution [26].

Recent research has explored the potential of high-density polyethylene (HDPE) and polypropylene (PP) in the manufacturing of geogrids for slope stabilization [27,28]. Various researchers have highlighted the favorable properties of these materials, such as their high tensile stiffness and resistance to elongation [29,30]. These characteristics allow the geogrids to support large tensile loads, contributing significantly to the stability of slopes. Furthermore, the open structure design enables efficient interlocking with natural fill material, forming a structurally stable embankment, comparable in function to a gravity wall [31,32,33,34].

High-density polyethylene (HDPE) and polypropylene (PP) are polymers widely used in the plastics industry, each with unique characteristics that define their applications and impact on the environment. This structure gives it exceptional chemical resistance, low water absorption, good tensile strength, and a stiffness that grants durability and impact resistance [35]. HDPE is found in a variety of applications, from food and beverage containers to water and gas pipes, as well as in geomembranes. However, HDPE waste represents a significant environmental problem due to its resistance to degradation, contributing to accumulation in landfills and environmental pollution [36].

On the other hand, PP, formed by the polymerization of propylene, has a lower density compared to HDPE, making it lighter. This polymer is known for its heat resistance, which allows its use in applications requiring sterilization, and is also resistant to fatigue [37]. PP is used in a variety of products, including food containers, automotive components, and textiles. Like HDPE, PP is resistant to degradation, posing challenges in waste management and its environmental impact [30].

The use of recycled HDPE and PP in the manufacturing of geogrids is a technical innovation that leverages the desirable properties of these materials while maintaining their tensile strength, durability, and chemical stability. This application is not only technically and structurally beneficial in geotechnical engineering projects, but also environmentally advantageous. Recycling these plastics helps minimize the accumulation of plastic waste in landfills and the environment, contributing to a circular economy and reducing the overall environmental impact of the products [38,39].

Efforts to use recycled plastics in the manufacture of geogrids are supported by numerous studies that emphasize the feasibility and environmental benefits of this practice [40,41,42]. Therefore, recent advances in geogrid technology represent an important step towards improving slope stabilization techniques in geotechnical engineering [43,44,45,46,47]. These advancements refer not only to the incorporation of new materials, but also to the adoption of innovative manufacturing techniques. Among these, 3D printing and high-frequency plastic welding stand out for their ability to drive efficiency in the production process of geogrids, especially those made from recycled plastics [48,49]. The use of geogrids made from recycled plastics is proving to be promising in the field of slope stabilization. These materials have shown remarkable ability to reduce the deformability of the cover soil, which is crucial for structural stability in geotechnical applications. However, despite these advancements, more research and monitoring are needed to comprehensively assess the long-term durability and effectiveness of these geogrids. It is crucial to compare them with traditional materials, not only in terms of technical performance, but also considering cost-effectiveness and environmental impact [50].

Initial studies are encouraging, as they suggest that recycled plastic geogrids can maintain their mechanical properties and performance similarly to geogrids made with virgin plastics. Nonetheless, deeper analysis is required to confirm the viability of recycled HDPE and PP geogrids in slope stabilization applications. Therefore, the main objective of this study is to design, develop, and evaluate a geogrid prototype manufactured from recycled thermoplastic materials, specifically high-density polyethylene (HDPE) and polypropylene (PP), for slope stabilization. This encompasses the formulation and design of the geogrid, the physical and mechanical evaluation of its load-bearing capacities, both materials and the geometric composition of the geogrid, and the verification of its effective geomechanical interaction with the soil, taking this study towards a comprehensive understanding of the performance and applicability of these recycled thermoplastic geogrids in slope stabilization.

This study presents, for the first time, a well-detailed comparative analysis of the mechanical properties of five types of recycled PP/HDPE composites, showcasing their potential for several applications. The material exhibiting superior deformation capacity was pure recycled HDPE. It was selected for designing various mesh geometries (with rhombohedral- and trigonal-shaped apertures), structural thread configurations (braid, filament, and sheet), and welding techniques. Furthermore, using SolidWorks^®^ Simulation (Dassault Systèmes, Vélizy-Villacoublay, Francia), we evaluated the suitability of this material for a specific case study involving slope stabilization. Subsequently, a laboratory-scale prototype geogrid was successfully constructed by the extrusion process. In summary, this study offers a perspective to reducing the dependence on new plastic materials and taking advantage of existing waste, promoting the use of environmentally friendly practices in geotechnical engineering.

## 2. Materials and Methods

In this study, a methodology previously described by other authors was adopted, with some adaptations [51]. The recycling process of PP (polypropylene) and HDPE (high-density polyethylene) plastics to create sustainable geogrids included several stages: collection, pretreatment, and transformation of thermoplastic containers. The PP and HDPE containers (e.g., food, cleaning products, and other domestic packaging items) were collected at a recycling center, and identified and classified according to the Möbius triangle, with number 2 for HDPE and number 5 for PP [52]. Once classified, the plastics underwent a cleaning process that included the removal of labels and caps, followed by washing and rinsing to remove dirt and organic matter. They were then air dried for 2 to 3 days on a cloth or adsorbent paper. The next step was to crush the plastics in a shear mill, thus reducing the size of the material to facilitate its handling in the extrusion process. Each ground polymer was mixed separately until a homogeneous mixture was achieved using a high-speed mixer.

The recycled plastics treatment stage involved preparing five types of PP/HDPE combinations (0/100, 25/75, 50/50, 75/25, and 100/0% *w*/*w*), with a total mass of 300 g for each combination. These mixtures were then subjected to an extrusion process in a 25 mm diameter and 400 mm length single-screw extruder with an INVT frequency inverter, Model GOODDRIVE 10, laboratory type, where an average operating temperature of 195 °C was established, with a processing speed of 20–23.5 rpm.

To evaluate the mechanical properties of the resulting PP/HDPE polymer composites, tensile and flexural tests were performed using a SHIMADZU AGX-V universal testing machine with a 5 kN load cell, following ASTM D638-03 [53] (Type IV) and ASTM 790 [54] standards [55]. The tests allowed us to determine mechanical properties of the composites, such as elastic modulus, maximum stress, and maximum deformation. To better understand the mechanical properties of the resulting PP/HDPE polymer composites, five specimens were tested under controlled conditions. This included tensile and flexural tests performed using a SHIMADZU AGX-V universal testing machine with a 5 kN load cell, following ASTM D638-03 (Type IV) and ASTM 790 standards [55]. Each specimen was securely positioned within the grips of the testing machine to ensure uniform application of force and to prevent slippage. The data acquired from these tests included tensile stress, load, strain, displacement, and the elastic (Young’s) modulus, all of which were meticulously recorded in an experimental database.

Furthermore, the flexural properties of each composite variant were assessed using the same universal testing machine, although employing a different fixture/template designed for flexural testing. Preparation and testing of the specimens adhered to a three-point bending methodology as prescribed by ASTM D790-03 [54]. The tests utilized a span-to-depth ratio of 16:1. Following the determination of specimen length, alignment on the three-point bending apparatus was executed. The flexural tests proceeded at an average speed of 1.35 mm/min, with the support span adjusted to range from 45.10 to 55.06 mm.

The ratio of crosshead speed and deflection were quantitatively analyzed using the following equations [56]:

(1) $$R=\frac{ZL^{2}}{6d}$$

(2) $$D=\frac{rL^{2}}{6d}$$

where R signifies the crosshead speed, L denotes the length of the specimen in millimeters, d represents the depth of the beam in millimeters, Z indicates the rate of deformation of the specimens (0.01 mm/mm/min), and r is the deformation (0.05 mm/mm).

In the geogrid design phase, a comprehensive evaluation of the mechanical properties of the five PP/HDPE composites was carried out to determine the most suitable material for the formation of the geogrid. Likewise, different mesh geometries (with a rhombohedral- and trigonal-shaped aperture), configurations of the structural threads (braid, filament, and sheet), and types of welding were evaluated. The selected designs were chosen based on their performance in preliminary mechanical tests and their potential for effective soil interaction.

Hot air welding was carried out for joining the polymeric strands due to its adaptability and efficiency in producing strong reliable joints. The welding parameters were carefully optimized by conducting a series of experiments that varied both the temperature and the duration of the welding process. Temperatures were set at 320, 330, 350, and 360 °C to determine the optimal conditions that yielded the strongest welds without compromising the integrity of the PP/HDPE composite material. The duration of the welding process was also varied, ranging from 15 to 80 s, to evaluate the effect of time on the quality and resistance of the welded joints.

Additionally, the determination of the melting point of the polymer composites was conducted using the Fisher-Johns melting point apparatus, serial 4022. To ensure consistency and reproducibility of the results, the heating rate of the apparatus was set to 2 °C per minute.

Stability criteria for slopes were established in accordance with the Ecuadorian Construction Standard (NEC-15) [57], and mechanical models of slope strips were created using the Morgenstern–Price and Spencer methods [58,59]. The analysis was performed with GeoStru 4 software (GeoStru Company, Cluj-Napoca, Romania), comparing the mechanical properties of different types of geogrids. In addition, SolidWorks Simulation software SP2.1 (Dassault Systèmes, Vélizy-Villacoublay, France) was used for linear static finite element analysis of the mechanical behavior of geogrids.

Finally, different analyses and tests were conducted to evaluate the resistance and behavior of the geogrid, using a universal testing machine. For tensile tests, standards such as ASTM D6637 and ASTM D4595 [60] were considered, recording load and displacement data with Bluehill 2 software (Instron Corporation, Norwood, MA, USA). Through these tests, aspects such as the rigidity and elastic behavior of the geogrid were evaluated.

The data were obtained directly in Excel spreadsheet format and migrated to SPSS (Statistics Package for the Social Sciences, version 21, SPSS Inc., Chicago, IL, USA), as needed. The SPSS statistical program was used to compare the different variables using ANOVA and Tukey tests. In retrospective comparisons of paired data, it is preferred to use Tukey’s HSD (honestly significant difference) test [61].

## 3. Results

### 3.1. Evaluation of Specimens

#### 3.1.1. Tensile Test

The tensile properties of the composites, encompassing tensile modulus, tensile strength, and maximum deformation, are summarized in Table 1. This table delineates the mechanical resilience and elasticity of the specimens under uniaxial tension.

The ANOVA analysis carried out on the tensile properties of the mixtures between PP and HDPE shows a significant difference between groups of 0.000 with a confidence value of 95%. The Tukey statistical test was also applied with an alpha value of 0.05, determining four different groups: (a) T1, (b) T2, (c) T3 and T4, and (d) T5. The ANOVA analysis is presented in the Supplementary Materials.

**Figure 1:**
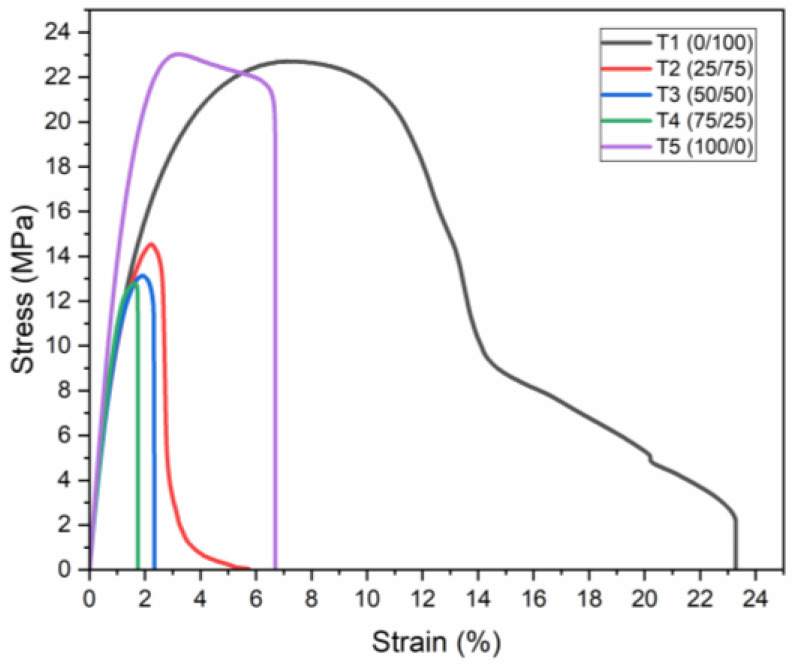
Stress–strain curve of tensile tests.

Furthermore, the stress–strain curves, represented in Figure 1, exhibit the elastic and plastic deformation behaviors of the composites under applied stress, highlighting the response of the materials to tensile loads.

Initial observations from Table 1 reveal a 4% reduction in the elastic modulus of the composite containing 25% PP compared to pure recycled HDPE, indicating an initial decrease in stiffness. With increasing PP content (50% *w*/*w* and above), a noticeable enhancement in the elastic modulus was observed, suggesting that PP acts as a reinforcement within the HDPE matrix. From these results, it is evident that the T5 composite (100% PP) demonstrated the highest increase in elastic modulus, approximately 32% higher than that of the T1 (100% HDPE) composite. The T4 composite (75/25 PP/HDPE) also exhibited significant elasticity, surpassing the T2 composite (25/75 PP/HDPE) by 13%. These results are consistent with those reported by Noor Hasanah et al. (2014), who analyzed the mechanical properties of seven different rPP/rHDPE blends. Their findings indicated that the modulus of elasticity increased by more than 90% with an increase in rPP content within the rPP/rHDPE blends [62].

On the other hand, Table 1 indicates that the maximum tensile strength was achieved by the T5 composite, with the T1 composite showing slightly lower strength by 2%. This suggests that the pure PP matrix has a higher tensile strength compared to composites with HDPE. This result indicates that the incorporation of PP into the HDPE matrix resulted in a decrease in tensile strength, as evidenced by the 36% reduction in the T2 composite compared to the T1 composite. Conversely, adding HDPE to the PP matrix led to a more significant reduction in tensile strength, as seen in the T4 composite.

Finally, the results of Table 1 indicate that the T1 composite exhibited the highest resistance to deformation under tensile load, indicative of HDPE’s ductile nature [63]. Conversely, PP’s inclusion tends to reduce the material’s plasticity, making it more prone to deformation at lower loads due to its inherent brittleness.

The experimental data from this test reveal a complex interplay between the addition of PP and HDPE, affecting the tensile properties of the composites. The addition of PP to HDPE generally enhances the elastic modulus but reduces tensile strength and plasticity, indicating a compromise between stiffness and ductility. These findings are essential for the development of PP/HDPE composites with tailored mechanical properties for specific applications, considering the inherent material compatibility challenges.

#### 3.1.2. Flexural Test

The flexural behavior of the composites was similarly analyzed, with results presented in Table 2. These tests evaluated the resistance of materials to deformation under bending forces.

**Figure 2:**
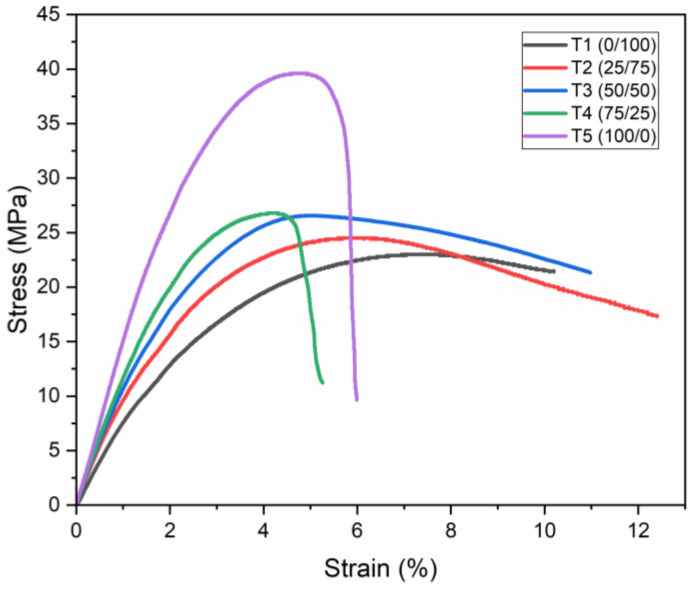
Stress–strain curve of flexural tests.

Furthermore, the stress–strain curves of the composites in the flexural tests are shown in Figure 2. The ANOVA analysis carried out on the flexural properties of the mixtures between PP and HDPE show a significant difference between groups of 0.000 with a confidence value of 95%. The Tukey statistical test was also applied with an alpha value of 0.05, determining three different groups: (a) T1, (b) T2 and T5, and (c) T3 and T4.

The stress–strain curves for the flexural tests, depicted in Figure 2, provide insights into the resistance of composites to bending stresses. These results indicate that the flexural modulus and strength generally increase with the PP content, with T5 showing the highest resistance to deformation.

From Table 2 we can compare the flexural modulus and flexural strength, which exhibit the superior mechanical strength and elasticity of the T5 composite over others. This enhancement in properties is attributed to the crystalline nature of PP, which strengthens intermolecular bonding, thus elevating the composite’s resistance and deformation capabilities. These results are consistent with those reported by Lin et al., 2015, who investigated the influence of HDPE content on the mechanical properties of PP/HDPE composites, where they found that both the modulus and flexural strength are higher when there is a higher content of PP in the polymer blend [64].

The interaction between PP and HDPE within the composite matrix significantly influences the ductility of the material, as observed in the deformation results (Table 2). The addition of PP reduces the plasticity of the HDPE matrix, making it more brittle and less ductile under load. This behavior suggests a complex relationship between the two polymers, possibly due to their inherent miscibility issues, leading to phase separation and differential mechanical behaviors. Since T5 is the specimen that presents a maximum deformation point, pure HDPE was chosen for the simulation of the geogrid.

The results of tensile and flexural tests illustrate the complex effects of the incorporation of PP into HDPE on the mechanical properties of the composites. These findings contribute to a deeper understanding of the material science behind PP/HDPE composites, with implications for their application in geotechnical and other engineering domains.

#### 3.1.3. Strand Configuration

The study also explored three strand configurations: braid, filament, and sheet. The results for tensile tests for braid and filament configurations are shown in Table 3.

**Figure 3:**
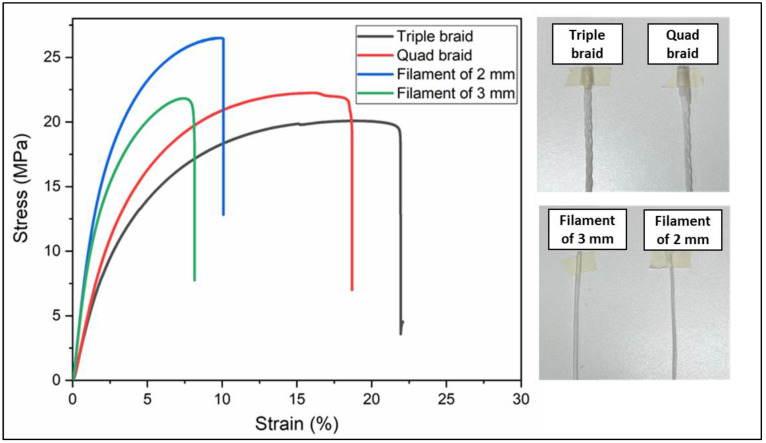
Tensile stress–strain curve plot of geogrid configurations.

It should be noted that the braids provide greater deformation during a tensile stress, as demonstrated in Figure 3.

**Figure 4:**
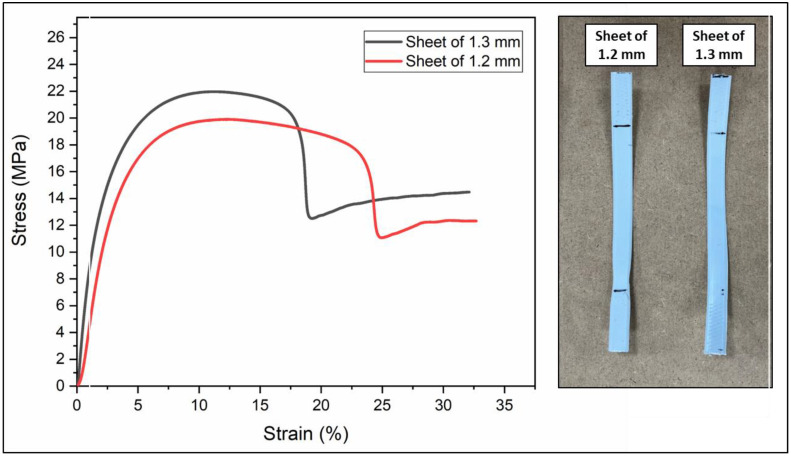
Force–displacement graph of sheet tensile tests.

It should be noted from the mechanical behavior of the samples that the filaments have better tensile strength than the braids; however, they have fragile behavior. Figure 4 also shows that the 1.3 mm sheet has greater mechanical behavior than the 1.2 mm thick sheet. The fact that its tensile strength is comparable to that of the filaments shown in Figure 3 is highlighted, adding ductile behavior, which is why the 1.3 mm sheet was chosen to obtain the geogrid prototype.

The ANOVA analysis carried out on the tension properties of the braid and filament configurations shows a significant difference between groups of 0.000 with a confidence value of 95%. The Tukey statistical test was also applied with an alpha value of 0.05, determining two different groups: (a) filament of 3 mm and filament of 2 mm, and (b) quad braid and triple braid.

The sheet configuration tests (Table 4) showed that a 1.3 mm thickness provided more consistent mechanical behavior compared to a 1.2 mm thickness.

The ANOVA analysis carried out on the tension properties of the sheet configuration shows a significant difference between groups of 0.000 with a confidence value of 95%.

Figure 4 also shows that the 1.3 mm sheet presents a greater mechanical behavior than the 1.2 mm thick sheet. The fact that its tensile strength is comparable to that of the filaments shown in Figure 3 is highlighted, adding ductile behavior, which is why the 1.3 mm sheet was chosen to obtain the geogrid prototype.

#### 3.1.4. Welding Evaluation

The efficacy of external heating using hot air for welding was assessed, with welds performed at various temperatures and durations (Table 5). Additionally, the melting temperature of the composites was determined to be 125 °C, providing a crucial parameter for optimizing the welding process.

**Figure 5:**
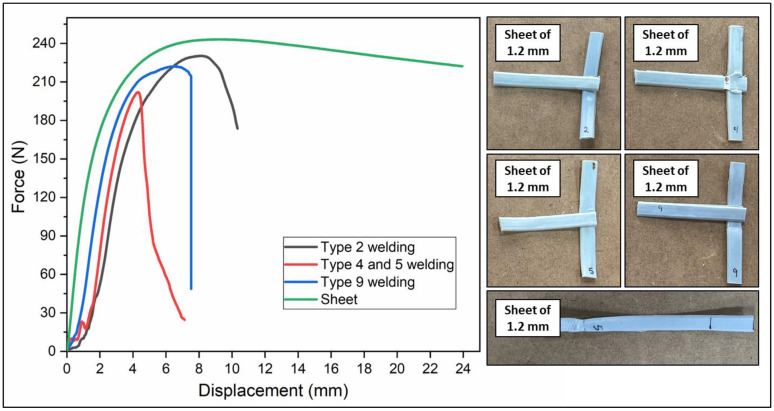
Force–displacement graph of welding tensile tests.

The force–displacement plot (Figure 5) indicates that welding times between 20 and 30 s and a temperature of 350 °C offered an optimal balance between efficiency and mechanical integrity.

### 3.2. Geogrid Prototypes

#### 3.2.1. Design and Evaluation

**Figure 6:**
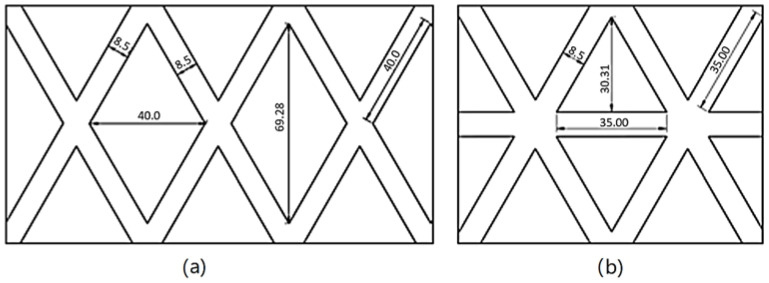
Geogrid designs. (**a**) Rhombohedral shape. (**b**) Trigonal shape.

In this study, two geogrid designs, with a rhombohedral- and trigonal-shaped aperture, were proposed, with their dimensions detailed in Figure 6. In section (a), the rhombohedral design is presented, which shows an aperture determined by the diagonals of the rhombus, with an aspect ratio of 1.73. Likewise, section (b) illustrates the design in trigonal form, where the aspect ratio of the aperture is calculated based on the base and height of the base triangle, reaching a value of 0.87.

**Figure 7:**
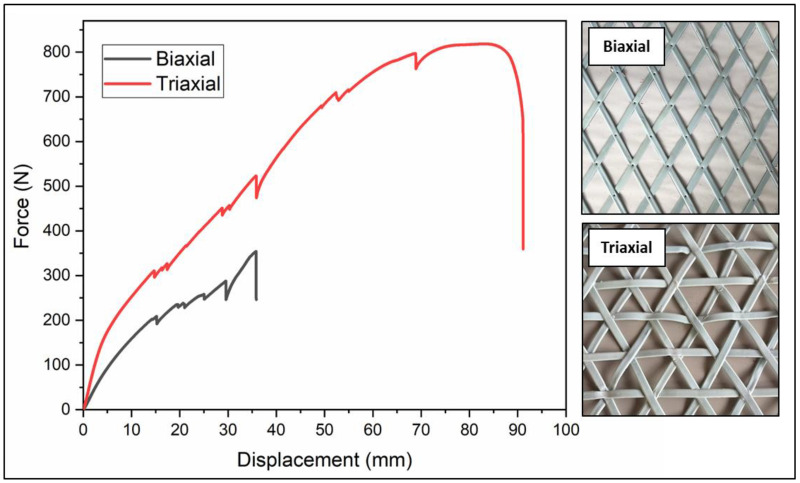
Force–displacement graph of geogrid tensile tests.

Tensile tests revealed the superior mechanical performance of the trigonal geogrid over the rhombohedral design, demonstrating greater load support and ductility, as shown in Table 6 and Figure 7.

The ANOVA analysis carried out on the tension properties of the geogrid types shows a significant difference between groups of 0.000 for the elastic limit strength and 0.011 for the maximum strength, with a confidence value of 95% in both cases.

**Figure 8:**
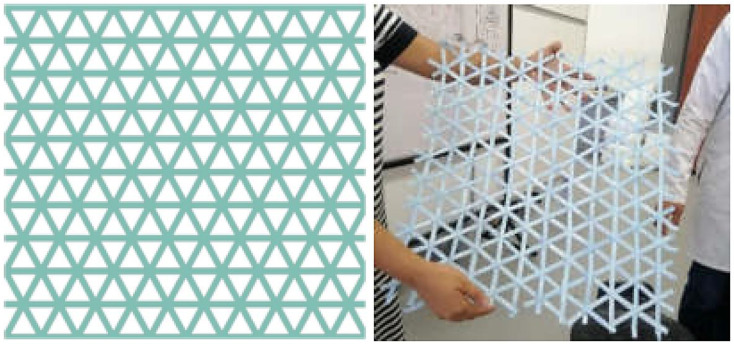
Final prototype of the trigonal geogrid.

The selection of 1.3 mm sheets and a welding process at a temperature of 350 °C for 20 to 30 s for constructing the geogrid prototypes was based on a carefully considered compromise between tensile strength and the material’s ductility. These conditions were meticulously chosen after evaluating how to maximize the efficacy of geogrids in slope stabilization applications, where both strength and flexibility are crucial. The thickness of 1.3 mm provides sufficient robustness to withstand the forces exerted during tension without sacrificing the material’s ability to undergo controlled deformation and adapt to terrain variations. On the other hand, welding at 350 °C for a period of 20 to 30 s was identified as the optimal range for creating durable and homogeneous connections between the ribs, essential for the structural integrity of the geogrids under load. When evaluating the rhombohedral and trigonal geometries under these specific manufacturing conditions, the superiority of the trigonal design was demonstrated, which not only favors a more uniform load distribution, but also enhances interaction with the soil, resulting in greater slope stability. This balance between tensile strength and ductility, along with the optimized geometric configuration, underscores the importance of a careful selection of design and manufacturing parameters to enhance the effectiveness of geogrids, paving the way for the development of innovative and sustainable geotechnical solutions. From these results, to manufacture the geogrid prototype, the geometry of the trigonal geogrid was chosen, since it has superior mechanical behavior than the rhombohedral geogrid. Figure 8 shows the prototype of the trigonal geogrid.

#### 3.2.2. Simulations of Slope Profile

Firstly, to assess the load-bearing capacity, the total active earth pressure (P_AE_) was calculated using Equation (3) [65]:

(3) $$P_{AE}=\frac{1}{2}\times \gamma \times H^{2}\times K_{v}\times K_{AE}$$

where γ is the unit weight of the fill soil, H is the height of the retaining wall, K_v_ is the vertical seismic coefficient, and K_AE_ is the dynamic active thrust coefficient.

The dynamic active thrust coefficient was obtained from the Equation (4), where the value of Ψ can be calculated by Equation (5) [65]:

(4) $$K_{AE}=\frac{{cos}^{2}\times \left(\varphi -\theta -\psi \right)}{cos\psi \times {cos}^{2}\theta \times cos\left(\delta +\psi +\theta \right)\times {\left[1+\sqrt{\frac{sin\left(\varphi +\delta \right)\times sin\left(\varphi -\beta -\psi \right)}{cos\left(\delta +\psi +\theta \right)\times cos(\beta -\theta )}}\right]}^{2}}$$

(5) $$\psi ={tan}^{-1}\left(\frac{K_{h}}{1-K_{v}}\right)$$

where φ is the angle of internal friction of soil, δ is the angle of wall friction, β is the backfill angle, θ is the slope of back wall to the vertical, K_h_ is the horizontal earthquake coefficient, and K_v_ is vertical earthquake coefficient. For slope stability analysis, the safety factor was determined considering variable loads, in accordance with NEC-SE-CG Loads (Non-Seismic) regulations, geotechnical soil characteristics as outlined in Table 7, and adherence to NEC-SE-GC Geotechnics and Foundations regulations with a safety factor of 1.5 for minimum design cuts [66]. This factor is crucial given the significant housing and vehicle load in the case study.

**Figure 9:**
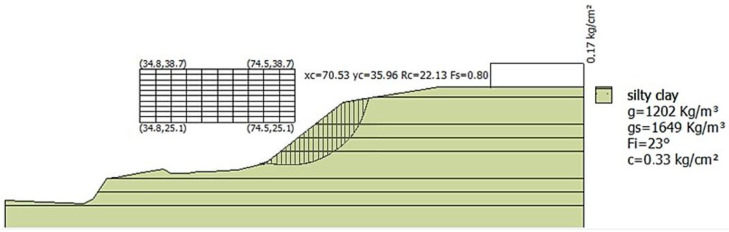
Simulated slope profile in GEO5 Geotechnical Software.

Slope profiles were analyzed using mechanical strip models via the Morgenstern–Price and Spencer methods [67], simulated with GeoStrud 4 software, as depicted in Figure 9. Profile 1 exhibited the lowest stability, with a safety factor below 1, indicating instability.

The results show that profile 1 is the least stable compared to profiles 2 and 3, therefore being the most susceptible. The values of the safety factors obtained for profiles 1, 2, and 3 were 0.80, 1.37, and 0.88, respectively. The NEC regulations establish that a safety factor greater than or equal to 1 indicates stability, while a safety factor less than or equal to 1 indicates instability. In this case, profile 1 has a safety factor of 0.80, indicating instability.

According to the limit equilibrium method of Morgenstern–Price and Spencer (simulated on GEO5 Geotechnical Software), it was found that the factor of safety for the slope was increased by 51.8% with the reinforcement of a trigonal-shaped geogrid, compared to the silty clay soil slope without any reinforcement.

#### 3.2.3. Simulation of Geogrids

This study examines the mechanical behavior of two types of geogrids (with a rhombohedral- and trigonal-shaped aperture) via computational simulations conducted using SolidWorks Simulation software SP2.1, where the von Mises stress analysis was conducted using the numerical method.

The dimensions of the specimens designed for simulation were 359 mm in length, 200 mm in width, and 1.3 mm in thickness; these parameters mirror the dimensions of geosynthetic specimens used in experimental tensile tests. The material chosen for the simulation was recycled HDPE, based on previous experimental outcomes. The mechanical properties of this material are detailed in Table 8, corresponding to those of a material processed by injection molding.

The average density obtained from three samples testing of recycled HDPE was 0.940 ± 0.021 g/cm^3^. The result presents a percentage difference of 3% compared to 0.97 g/cm^3^ density for post-consumer HDPE selectively chosen from recycled detergent bottles [68]. This scenario repeats itself again, with a density lower by 3% than the theoretical value of 0.942 g/cm^3^ for recycled HDPE used in a second study [69].

**Figure 10:**
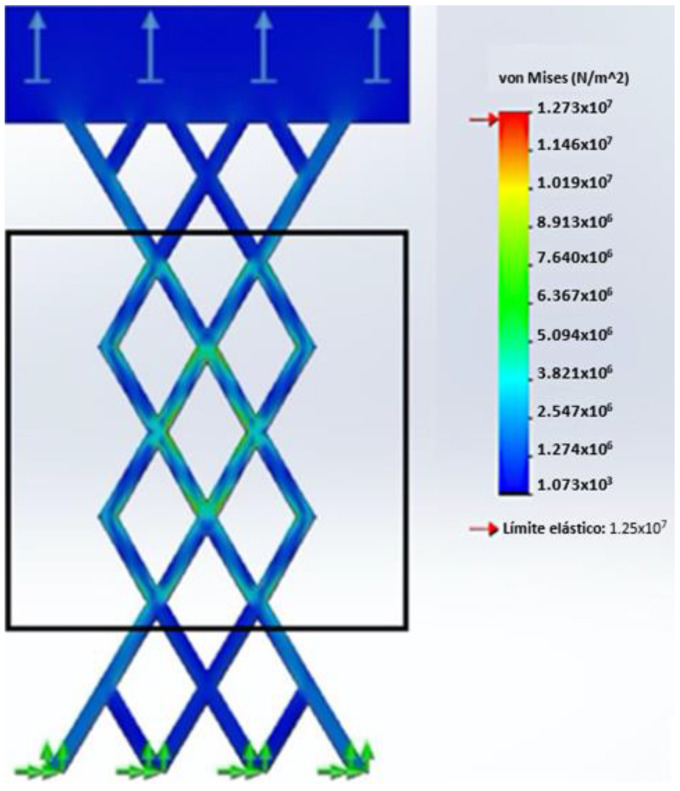
Static stress analysis of rhombohedral geogrid.

To simulate the tensile test of the rhombohedral geogrid, movement at the lower ends of the specimen was restricted, with lateral ribs near the central node kept together. Additionally, a top support sheet was incorporated to simulate the uniform application of tensile stress. The load-bearing capacity of the geogrid is determined by the yield limit of the material, which is a critical factor for selecting the optimum geogrid geometry. Using a reference value for the yield limit of recycled HDPE of 12.51 MPa, the simulation indicated that the central node of the specimen supports a maximum force of approximately 104 N before undergoing permanent deformation. The simulation observations, depicted in Figure 10, show failure in the midsection of the specimen, with high-stress areas represented in red. The boundary conditions of the present study included fixed restraints in all directions along bottom edges of the geogrid geometry to mimic universal testing machine anchorage, and, at the other end, uniform loading was applied at the top edge, as previously mentioned.

**Figure 11:**
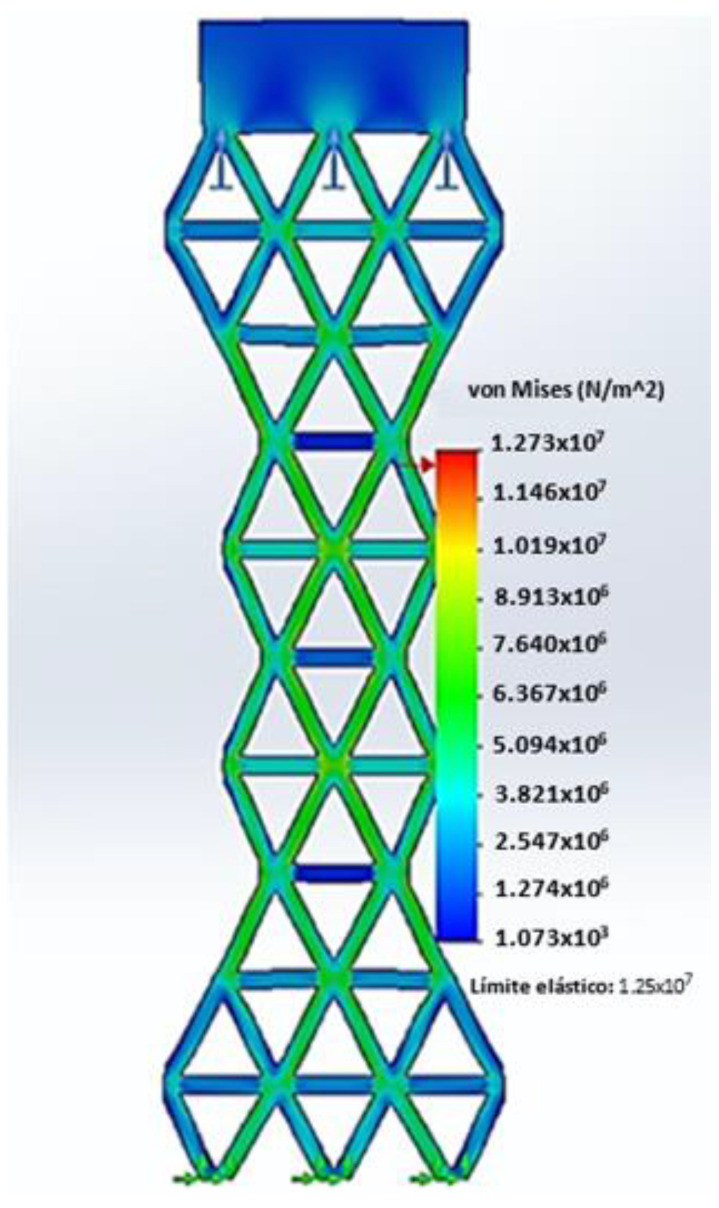
Static stress analysis of trigonal geogrid.

In contrast, Figure 11 demonstrates how the inclusion of a transverse rib, characteristic of a trigonal geogrid, increases the maximum load resistance to 210 N, showcasing a significant improvement in mechanical behavior by providing a broader range of force before reaching its yield limit.

In the context of the von Mises analysis, the value of the safety factor for both geogrids was greater than one. When the factor of safety is greater than one, it means that the material or structure is designed to withstand loads greater than those actually experienced under service conditions [70]. In this case, the material has a sufficient load capacity to resist the applied loads, indicating a safe and conservative design.

It is important to mention that for both geometric configurations, three mesh sizes were tested: fine, medium, and coarse. The comparison of the von Mises stress values between the three sizes showed an average difference of 3.9%. Furthermore, to check the quality of the tested meshes, the aspect ratio was determined in the SolidWorks software SP2.1. According to the software specifications, a good quality mesh has an aspect ratio of less than 5 for more than 90% of its elements. The results of this study showed that all the meshes tested met the quality criteria for more than 98% of the elements.

The comparison of results between simulations and tensile tests conducted with the universal testing machine (UTM) is presented in Table 9. It is noted that the maximum resistance of 104 N of the rhombohedral geogrid in the simulation is 26% lower than the load supported in the UTM tests. Furthermore, a 10% error is identified in the resistance of a triangular geometry geogrid simulated in SolidWorks compared to 188.62 N supported during the UTM tests. This analysis reveals that the maximum tension without inducing permanent deformations is achieved with a trigonal geogrid.

Differences between both evaluation methods can be attributed to displacements in the joining nodes of the geogrid during the tensile test in a universal testing machine. It is acknowledged that the node design in the software exhibits greater rigidity compared to real testing conditions, which could explain the observed variations in resistance and displacement properties.

The geometry of the trigonal geogrid was chosen since it has better mechanical behavior; in mechanical tests, it has greater rigidity, is more uniform in all directions, is more resistant, and has a lower probability of deformation when subjected to external forces, in contrast to a rhombohedral geogrid.

## 4. Discussion

### 4.1. Preparation of Evaluation Specimens

The results obtained from the tensile and flexural tests of polypropylene/high-density polyethylene (PP/HDPE) composites demonstrate an interesting interaction between the composition of the materials and their mechanical properties, which is important for understanding the behavior of these composites in geotechnical and civil engineering applications. The data from this study reveal that the incorporation of PP improves the elastic modulus but decreases the tensile strength and plasticity of the composites, reflecting a balance between stiffness and ductility. This effect is attributed to the fact that PP, when added to HDPE, increases the material’s stiffness, making it less susceptible to deformation under tensile loads while also making it more brittle, decreasing its ability to elongate without fracture. These results are consistent with previous studies that have shown that the incorporation of PP could act as a reinforcement in polymer matrices, thus modifying the mechanical properties of the final composites. The decrease in maximum deformation with the increase in PP content in the composites can be attributed to the more rigid nature of PP compared to HDPE, which limits the material’s deformation capacity.

Furthermore, in the flexural test, it is evident that the interaction between PP and HDPE within the composite matrix significantly affects the material’s ductility, with the addition of PP reducing the plasticity of the HDPE matrix and making it more brittle. The improvement in the flexural modulus and strength with the increase in PP content in the composites suggests that PP can strengthen the intermolecular bonds and increase the material’s resistance to flexural forces. These results are consistent with previous research that has demonstrated that the incorporation of PP can improve the mechanical properties of HDPE composites, especially in terms of flexural resistance.

As can be seen, the interaction between PP and HDPE within the composite matrix has a significant impact on the material’s mechanical properties. The addition of PP in the HDPE matrix results in an increase in stiffness and a reduction in the propensity for deformation under tensile loads. These results suggest a trade-off between stiffness and ductility in the PP/HDPE composites. This behavior underscores the complexity of the relationship between both polymers and the material compatibility challenges that must be addressed for the development of PP/HDPE composites with specific mechanical properties. Therefore, the results of this study highlight the importance of careful composite design to balance the properties of individual components to meet specific application requirements, considering polymer interaction and material compatibility challenges.

In addition to mechanical properties, this study also examined other factors such as the strand configuration in polypropylene/high-density polyethylene (PP/HDPE) composites, with special attention paid to its influence on the properties of geogrids. The strand configuration refers to the arrangement and geometry of the structural filaments in the geogrid, which can adopt various patterns such as square, hexagonal, or triangular. It was found that this configuration significantly impacts the overall performance of the geogrid.

In this study, three strand configurations were evaluated—braided, filament, and sheet—to determine their impact on the tensile and flexural properties. Therefore, the findings suggest that both the strength and deformability of the geogrids could be conditioned by the strand configuration used in their manufacture. Specifically, the braided configuration confers superior stiffness and tensile strength compared to the other arrangements. These results, consistent with observations in tensile properties, emphasize the predominant influence of the strand structure on the mechanical properties of the composites, both tensile and flexural. Additionally, it was found that the thickness of the sheet affects the mechanical properties, showing a more uniform behavior in sheets of 1.3 mm compared to those of 1.2 mm. This difference is attributed to the distribution of stresses along the strands in each configuration, where the braided arrangement increases the number of contact points between the strands, thus facilitating load transfer and improving the stiffness and flexural resistance of the geogrids.

The analysis extended to the study of geogrid welding, evaluating the effectiveness of external heating through hot air. Tests were conducted at various temperatures and durations, determining that welding times between 20 and 30 s at 350 °C provide an optimal balance between efficiency and the mechanical integrity of the joints. This suggests that the duration and temperature of welding has a significant impact on the strength and quality of the welded joints in polymer geotextiles. The force–displacement curve (Figure 5) shows how different welding conditions affect the strength and integrity of the welded joints, highlighting the importance of appropriately selecting welding parameters to ensure the quality of the joints in geogrids.

Comparing our findings with the existing literature reveals congruence with prior studies. For instance, Zou et al. (2016) investigated the creep behavior of high-density polyethylene geogrids and highlighted the significance of geogrid properties in reinforced soil retaining walls. Our study complements this by elucidating the influence of PP/HDPE composite composition and geogrid design on mechanical behavior [71]. Similarly, Wang et al. (2015) explored the properties of glass fiber-reinforced plastic geogrids, emphasizing their mechanical strength and stability. While our study focuses on PP/HDPE composites, both investigations underscore the importance of material properties in geogrid performance [72]. Additionally, Ezzein et al. (2014) developed models to predict the load–deformation behavior of polypropylene geogrids under constant rate-of-strain loading. Our findings corroborate the importance of understanding geogrid mechanical behavior, albeit in the context of PP/HDPE [73].

### 4.2. Geogrid Prototypes

Slope stability analysis is an important aspect of geotechnical engineering that requires a detailed understanding of the mechanical properties of geogrids and the characteristics of the surrounding soil. Among these properties, the modulus of elasticity and tensile strength are fundamental for assessing the ability of geogrids to resist elastic deformations and support the stresses generated by the soil, ensuring their structural integrity.

Bending resistance also becomes important, as slopes are exposed to forces in both horizontal and vertical directions, requiring an effective distribution of these loads by the geogrid. Additionally, the geogrid–soil interaction, influenced by the friction angle between them and the load transfer capacity, is essential in slope stability analysis. This approach allows for the determination of the suitability of the geogrid to strengthen slope stability and prevent landslides or collapses. The results of this analysis enable determining of whether the geogrid can resist soil forces and maintain slope stability, which is fundamental for the successful design and implementation of stabilization measures in geotechnical projects.

In this study, a linear static finite element analysis of the mechanical behavior of geogrids in SolidWorks Simulation software SP2.1 using the von Mises maximum stress method was conducted. This analysis allowed the evaluation of the strength and load-bearing capacity of geogrids under simulated conditions, which is essential for understanding their performance in geotechnical applications.

In this study, the safety factor for the geogrids was greater than one, indicating that the material designed from extruded HDPE reaches its maximum load-bearing capacity right at the threshold where it begins to deform permanently, suggesting it can fully utilize its elasticity without permanent deformations.

A detailed comparison of the results between simulations and experimental tests reveals significant differences in the maximum strength of the geogrids, suggesting possible divergences in predicting the mechanical behavior of these materials. These disparities can be attributed to factors such as node design in simulation software and buckling.

The simulation indicated that the maximum strength of the rhombohedral geogrid is 26% lower than the strength measured experimentally in universal testing machine (UTM) tests, while for the trigonal geometry geogrid, the error in simulated strength compared to real tests was 10%. These discrepancies highlight the importance of validating simulations with experimental tests to improve the accuracy of simulation models.

Strength, load-bearing capacity, and modulus of elasticity are key elements in the geotechnical performance of geogrids. Thus, the geogrid made from extruded HDPE was shown to be capable of supporting applied loads without incurring significant permanent deformations, maintaining adequate elastic recovery. The flexural resistance of this material is also crucial, indicating its ability to resist bending without fractures or excessive deformations.

## 5. Conclusions

This study reveals how the incorporation of polypropylene (PP) into high-density polyethylene (HDPE) compounds significantly improves mechanical properties such as elasticity and tensile strength, highlighting the viability of using recycled materials in critical engineering applications. This approach not only reduces the consumption of virgin resources, but also promotes more sustainable construction practices, aligning with global efforts to combat the accumulation of plastic waste and mitigate climate change.

The manuscript emphasizes how increasing the PP content in HDPE compounds significantly alters both the modulus and tensile strength, which is crucial for geonet applications where mechanical strength is paramount. The mixture of PP with HDPE results in a composite material that offers a more efficient stress distribution and better resistance to deformation, which are essential qualities for soil stabilization and reinforcement. However, the research highlights the selection of pure recycled HDPE for the manufacture of geogrids, which was justified by its final results. The extrusion process used to create 1.3 mm thick laminated threads, welded with hot air, results in a geonet with excellent force distribution and tensile strength. The choice of a trigonal geometry for the geogrid is based on its superior mechanical behavior, offering improved stiffness, uniformity, and resistance, making it optimal for applications requiring high strength and stability.

The study addresses the importance of geometric and manufacturing configurations in the performance of geogrids, indicating how the trigonal-shaped aperture design surpasses the rhombohedral due to its advanced load distribution capabilities. It also emphasizes the relevance of selecting optimal welding conditions and thread configurations based on mechanical performance to enhance the durability and effectiveness of geonets in practical applications.

The implications of this study for the practice of geotechnical engineering are profound, providing a basis for the development of more effective, reliable, and customizable geosynthetics. This work underscores the possibility of adjusting materials and geonet designs to specifically meet environmental and load requirements, offering promising solutions to sustainable infrastructure challenges in the context of climate change.

Moreover, the research suggests that optimizing the proportions of PP and HDPE and geogrid configurations could achieve an optimal balance between stiffness and ductility, which is crucial for designing effective stabilization solutions. However, the need for further research to explore the long-term performance of these geonets under various environmental conditions, including their resistance to chemical and biological degradation and their behavior under cyclic and dynamic loads, is recognized.

Finally, this study not only enhances our understanding of PP/HDPE compounds and their application in geogrid technology, but also opens new avenues for the use of geosynthetics in the development of sustainable infrastructure. By integrating materials science with geotechnical engineering, it promotes the development of innovative solutions that improve the resilience and sustainability of built environments, marking a step forward toward more sustainable and responsible construction practices, and demonstrating the potential of recycled plastics in mitigating the negative environmental impacts associated with plastic production and waste.

## Figures and Tables

**Table 1:** Tensile properties of PP/HDPE composites.

Test Specimen Code (PP/HDPE)	Tensile Modulus (MPa)	Tensile Strength (MPa)	Maximum Deformation (%)
T1 (0/100)	1020 ± 21	22.46 ± 0.77	7.32 ± 0.57
T2 (25/75)	982 ± 45	14.48 ± 0.90	2.37 ± 0.28
T3 (50/50)	1040 ± 64	12.05 ± 1.04	1.49 ± 0.26
T4 (75/25)	1127 ± 51	12.82 ± 0.23	1.58 ± 0.14
T5 (100/0)	1499 ± 26	22.97 ± 0.63	2.98 ± 0.28

**Table 2:** Flexural properties of PP/HDPE composites.

Test Specimen Code (PP/HDPE)	Flexural Modulus (MPa)	Flexural Strength (MPa)	Maximum Deformation (%)
T1 (0/100)	595.13 ± 54.25	24.63 ± 1.07	7.95 ± 0.35
T2 (25/75)	718.31 ± 27.72	24.66 ± 1.04	6.19 ± 0.55
T3 (50/50)	941.40 ± 32.67	24.86 ± 1.13	4.74 ± 0.38
T4 (75/25)	1095.15 ± 45.25	25.80 ± 2.37	4.13 ± 1.00
T5 (100/0)	1583.79 ± 81.19	41.76 ± 1.81	5.30 ± 0.47

**Table 3:** Tensile properties of braid and filament configurations.

Configuration	Elastic Module (N)	Maximum Tension (MPa)	Maximum Deformation (mm)
Triple braid	360.96 ± 28.28	19.67 ± 1.68	15.83 ± 4.93
Quad braid	524.12 ± 39.66	22.04 ± 0.97	14.89 ± 1.33
Filament of 2 mm	903.64 ± 48.34	26.11 ± 0.63	8.85 ± 0.88
Filament of 3 mm	595.31 ± 74.98	23.25 ± 1.23	8.12 ± 0.94

**Table 4:** Tensile properties of sheet configuration.

Configuration	Elastic Limit in Strength (N)	Maximum Tension (MPa)	Maximum Deformation (mm)
Sheet of 1.2 mm	105.33 ± 2.55	18.16 ± 0.35	26.03 ± 0.58
Sheet of 1.3 mm	98.46 ± 2.08	21.77 ± 0.65	21.60 ± 0.78

**Table 5:** Weld tensile mechanical test results.

Type of Welding	Temperature (°C)	Time (s)	Maximum Stress (MPa)	Maximum Force (N)	Maximum Displacement (mm)
Type 1	320	40	14.94	165.08	2.67
Type 2		60	19.30	213.23	6.12
Type 3		80	17.24	190.52	4.69
Type 4 and 5	350	25	18.09	199.98	4.47
Type 6		15	9.62	106.35	1.54
Type 7	330	30	8.35	92.26	2.08
Type 8		20	8.40	92.86	3.11
Type 9		15	17.75	196.11	3.84
Type 10	360	15	7.58	83.79	1.62
Type 11		20	12.69	140.18	3.95
Type 12		19	21.37	236.16	6.14

**Table 6:** Tensile properties of rhombohedral and trigonal geogrids.

Geogrid Prototyping	Elastic Limit Strength (N)	Maximum Strength (N)	Maximum Displacement (mm)
Rhombohedral	139.83 ± 4.35	404.84 ± 88.52	45.74 ± 9.53
Trigonal	188.62 ± 17.57	620.21 ± 159.44	59.57 ± 14.03

**Table 7:** Slope statistics.

Stratum	Cohesion (Kg/cm^2^)	Undrained Cohesion (Kg/cm^2^)	Shear Resistance angle (°)	Specific Weight (Kg/m^3^)	Saturated Weight (Kg/m^3^)	Lithology
1	0.33	N/A	23	1202	1649	Silty clay

**Table 8:** Mechanical and physical properties of injection HDPE.

Elastic Limit in Tension (MPa)	Tensile Limit (MPa)	Elastic Module (MPa)	Density (g/cm^3^)
12.51	22.46	1020.24	0.94

**Table 9:** Results of tensile mechanical properties of geogrids.

Material	Rhombohedral		Trigonal	
	UTM	SolidWorks	UTM	SolidWorks
**Elastic Limit in Strong (N)**	139.83 ± 4.35	104	188.62 ± 15.71	210
**Error (%)**	26%		10%	

## Data Availability

Data are contained within the article.

## Supplementary Materials

The following supporting information can be downloaded at: https://www.mdpi.com/article/10.3390/polym16081151/s1, Table S1: ANOVA table for tensile properties of PP/HDPE composites; Table S2: Tukey test table for tensile properties of PP/HDPE composites; Table S3: ANOVA table for flexural properties of PP/HDPE composites; Table S4: Tukey test table for flexural properties of PP/HDPE composites; Table S5: ANOVA table for tensile properties of braid and filament configurations; Table S6: Tukey test table for tensile properties of braid and filament configurations; Table S7: ANOVA table for tensile properties of sheet configuration; Table S8: ANOVA table for tensile properties of rhombohedral and trigonal geogrids; Table S9: ANOVA table for tensile properties of rhombohedral and trigonal geogrids.
