# Supplementary material for: Evaluation of the Effectiveness of Geogrids Manufactured from Recycled Plastics for Slope Stabilization—A Case Study
Source: Polymers (Basel). 2024 Apr 19;16(8):1151. doi: 10.3390/polym16081151 (PMC11053962; doi:10.3390/polym16081151)
Supplement: Supplementary file 1 [file polymers-16-01151-s001.zip › polymers-2918283-supplementary.pdf]

polymers

MDPI

Supplementary Materials

Evaluation of the Effectiveness of Geogrids Manufactured from Recycled Plastics for Slope Stabilization—A Case Study

Lenin Vicuña [1], Ximena Jaramillo-Fierro [2,*], Paúl Eduardo Cuenca [1], Brenda Godoy-Paucar [3], Jorge Daniel Inga-Lafebre [1], José Luis Chávez [4], Juan Fernando García [2], Diana Guaya [2] and Juan Diego Febres [1]

1 Departamento de Producción, Facultad de Ciencias Exactas y Naturales, Universidad Técnica Particular de Loja, San Cayetano Alto, Loja 1101608, Ecuador; lvicuna@utpl.edu.ec (L.V.); pecuenca2@utpl.edu.ec (P.E.C.); jdinga@utpl.edu.ec (J.D.I.-L.); jdfebres@utpl.edu.ec (J.D.F.)
2 Departamento de Química, Facultad de Ciencias Exactas y Naturales, Universidad Técnica Particular de Loja, San Cayetano Alto, Loja 1101608, Ecuador; jfgarcia@utpl.edu.ec (J.F.G.); deguaya@utpl.edu.ec (D.G.)
3 Carrera de Ingeniería Industrial, Facultad de Ciencias Exactas y Naturales, Universidad Técnica Particular de Loja, San Cayetano Alto, Loja 1101608, Ecuador; bngodoy@utpl.edu.ec
4 Departamento de Ingeniería Civil, Facultad de Ingenierías y Arquitectura, Universidad Técnica Particular de Loja, San Cayetano Alto, Loja 1101608, Ecuador; jlchavez3@utpl.edu.ec
* Correspondence: xvjaramillo@utpl.edu.ec; Tel.: +593-7-3701444

Table S1 Anova table for tensile properties of PP/HDPE Composites

Maximum_Deformation_Ten

	Sum of Squares	df	Mean Square	F	Sig.
Between Groups	116.325	4	29.081	255.623	.000
Within Groups	2.275	20	.114		
Total	118.600	24			

Table S2 Tukey test table for tensile properties of PP/HDPE Composites

Maximum_Deformation_Ten

Tukey B[a]

Specimen_Code	N	Subset for alpha = 0.05			
		1	2	3	4
T3	5	1.4871			
T4	5	1.5838			
T2	5		2.3720		
T5	5			2.9833	
T1	5				7.3230

Means for groups in homogeneous subsets are displayed.

a. Uses Harmonic Mean Sample Size = 5.000.

Table S3 Anova table for flexural properties of PP/HDPE Composites

ANOVA

Maximum_Deformation_Flx

	Sum of Squares	df	Mean Square	F	Sig.
Between Groups	44.137	4	11.034	30.962	.000
Within Groups	7.128	20	.356		
Total	51.264	24			

Table S4 Tukey test table for flexural properties of PP/HDPE Composites

Maximum_Deformation_Flx

Tukey B[a]

Specimen_Code	N	Subset for alpha = 0.05			
		1	2	3	4
T4	5	4.1284			
T3	5	4.7386	4.7386		
T5	5		5.3036	5.3036	
T2	5			6.1872	
T1	5				7.9464

Means for groups in homogeneous subsets are displayed.

a. Uses Harmonic Mean Sample Size = 5.000.

Table S5 Anova table for tensile properties of braid and filament configurations

ANOVA

Maximum_Deformation

	Sum of Squares	df	Mean Square	F	Sig.
Between Groups	240.068	3	80.023	13.225	.000
Within Groups	96.811	16	6.051		
Total	336.879	19			

Table S6 Tukey test table for tensile properties of braid and filament configurations

Maximum_Deformation

Tukey HSD[a]

Configuration	N	Subset for alpha = 0.05	
		1	2
Filament of 3mm	5	8.1240	
Filament of 2mm	5	8.8520	
Quad braid	5		14.8960
Triple braid	5		15.8360
Sig.		.965	.929

Means for groups in homogeneous subsets are displayed.

a. Uses Harmonic Mean Sample Size = 5.000.

Table S7 Anova table for tensile properties of sheet configuration

ANOVA

Maximum_Deformation

	Sum of Squares	df	Mean Square	F	Sig.
Between Groups	49.062	1	49.062	131.457	.000
Within Groups	2.986	8	.373		
Total	52.048	9			

Table S8 Anova table for tensile properties of rhombohedral and trigonal geogrids

ANOVA

Elastic_Limit_Strength

	Sum of Squares	df	Mean Square	F	Sig.
Between Groups	5953.112	1	5953.112	47.002	.000
Within Groups	1013.252	8	126.656		
Total	6966.364	9			

Table S9 Anova table for tensile properties of rhombohedral and trigonal geogrids

ANOVA

Maximum_Strength

	Sum of Squares	df	Mean Square	F	Sig.
Between Groups	115962.746	1	115962.746	11.020	.011
Within Groups	84183.651	8	10522.956		
Total	200146.397	9			
